# Giant Cell Tumor With Secondary Aneurysmal Bone Cyst in the Left Distal Humerus: A Case Report

**DOI:** 10.7759/cureus.65507

**Published:** 2024-07-27

**Authors:** Yaser Alhulaimi, Khaled K AlAbbasi, Osama S AlShaya, Talal N Alrawaf, Nasser H Aldosari, Basem Zogel

**Affiliations:** 1 Department of Orthopedic Surgery, King Fahad Medical City, Riyadh, SAU; 2 Department of Reconstructive Orthopedics, King Fahad Medical City, Riyadh, SAU; 3 Faculty of Medicine, Jazan University, Jazan, SAU

**Keywords:** resection of gct, gct distal humerus, bone curettage, gct, distal humerus, aneurysmal bone cyst, giant cell tumor

## Abstract

Giant cell tumor (GCT) is a common benign aggressive tumor that mostly occurs in the proximal tibia, distal radius, and distal femur but is rarely seen in the distal region of the humerus. It originally presents between the ages of 30 and 50 with suddenly occurring pain. Treatment is generally curettage adjuvant treatment if necessary and reconstruction if required.

In our case report, we present the clinical and radiological findings, diagnosis, and management of a 33-year-old female patient with a giant cell tumor (GCT) accompanied by a secondary aneurysmal bone cyst (ABC) in the left distal humerus, where the patient experienced pain for many years without significant history of trauma. Upon clinical examination, the patient displayed tenderness over the medial side of the elbow but no noted swelling, redness, or hotness. She had a painless full range of motion, with an intact distal neurovascular examination. Imaging concluded GCT with secondary ABC. A biopsy confirmed the diagnosis, ruling out metastatic lesions. The patient underwent surgical intervention, with plate fixation, which yielded excellent outcomes.

## Introduction

Giant cell tumor (GCT) of the bone is a relatively common primary bone tumor, accounting for approximately 5% of all primary bone neoplasms [1,2]. While GCT can occur in various skeletal locations, it predominantly affects the epiphyseal regions of the long bones. However, the distal humerus represents an uncommon site for GCT occurrence, making such cases particularly noteworthy [3].

Adding to the rarity, the combination of GCT with a secondary aneurysmal bone cyst (ABC) presents an even more uncommon pathological entity [4]. This association, while recognized in the literature, is infrequently encountered in clinical practice, especially in atypical locations such as the distal humerus. The manifestation of an ABC within a GCT can further complicate the clinical presentation and handling of these lesions [5,6]. ABCs are blood-filled, cystic expansions of the bone that regularly form secondary to an underlying reason, such as a GCT. ABC formation within a GCT has been reported in up to 25% of cases [5,6].

Although GCTs in the distal humerus have been documented, they are relatively scarce, and the literature on their management is limited [6]. This is an essential query area, as the distal humerus is a pivotal anatomical location for elbow function and stability [7]. The successful handling of GCTs in this region is crucial for maintaining patient mobility and quality of life. A literature review revealed that only a handful of case reports and small case series have been published on GCTs of the distal humerus [6-8]. These studies have shown varying consequences, with some showing good practical results and low recurrence rates after curettage and bone grafting. In contrast, others have reported higher recurrence rates and the necessity for more extensive resection.

Furthermore, Zhen et al. conducted a retrospective analysis of the long-term outcomes of 132 patients with GCT treated with curettage and bone grafting and discovered an 18.2% recurrence rate [9]. Another study by Oda et al. monitored 68 GCT patients for an average of 11.1 years and found a 22.1% recurrence [10]. These studies highlight the significance of meticulous surgical planning and long-term follow-up for patients receiving GCTs, especially under challenging locations such as the distal humerus. In this report, we present a case of GCT with secondary ABC in the distal humerus, highlighting the diagnostic considerations, management approach, and outcomes of this unusual presentation. This case underscores the importance of considering rare pathological combinations even in atypical anatomical locations when evaluating bone tumors.

## Case presentation

In May 2023, a 33-year-old female, a known case of hypothyroidism on treatment, otherwise medically and surgically free with an adverse family history of malignancy, presented to our clinic with a complaint of progressive on/off left elbow pain that had started four years ago and was sometimes relieved by over-the-counter analgesia, without any history of trauma. The pain had recently worsened significantly, affecting her daily activities, particularly at night and while lifting objects. The patient sought medical attention at an outside hospital, where she underwent a full malignancy workup, which showed GCTs with ABC, and she decided to visit our clinic for further management and treatment. Physical examination revealed mild tenderness over the medial epicondyle with no noticeable swelling, redness, hotness, or compromised skin condition. The patient had a painless full range of motion in the left elbow joint, and the distal neurovascular examination was unremarkable. Chest radiography, chest computed tomography (CT), and blood test results were normal.

Radiological findings

**Figure 1:**
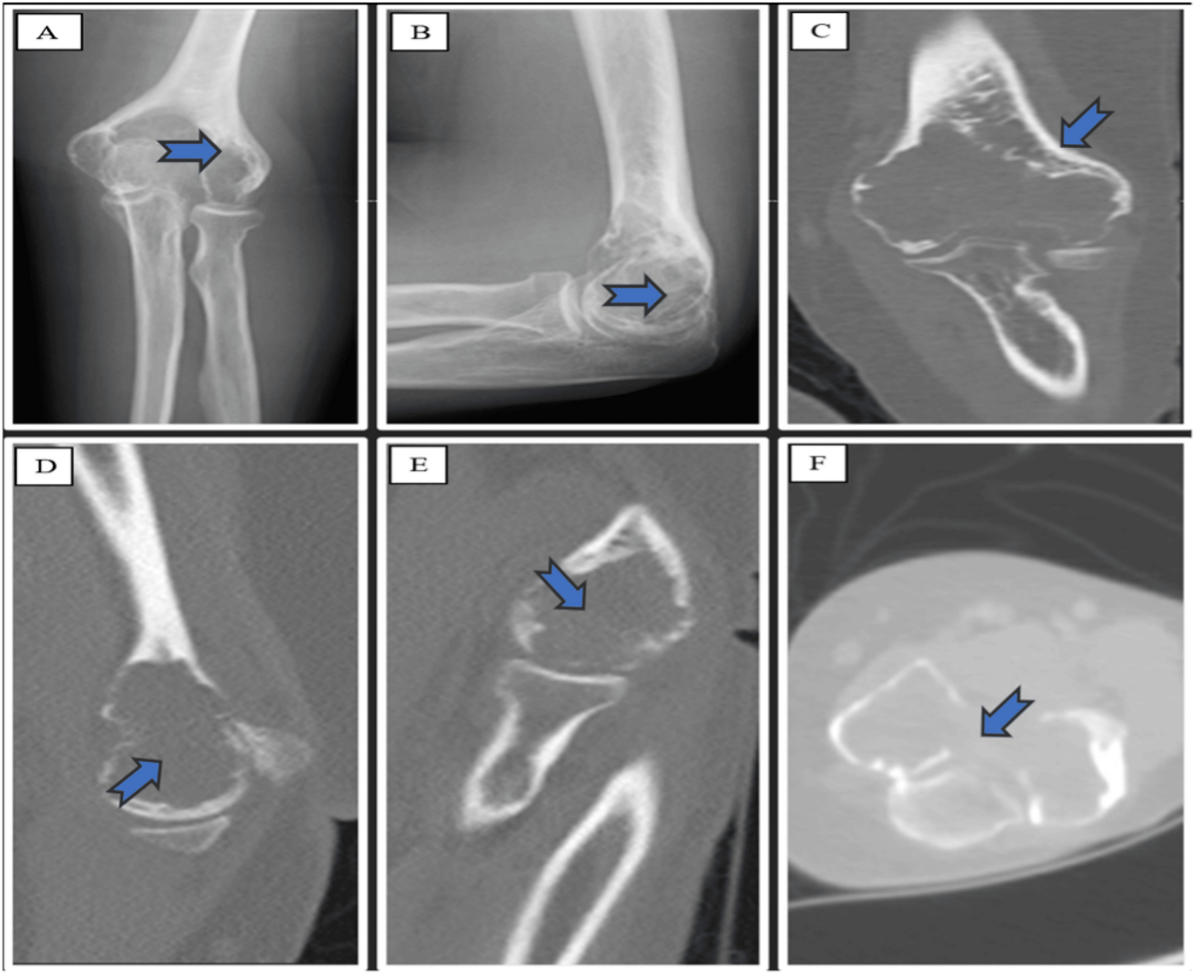
X-ray imaging and computed tomography (CT) of the left elbow revealed an epiphyseal expansile lytic lesion in the distal humerus. The X-ray and CT images of the left elbow provide a comprehensive view of the joint from various angles. The X-ray anterior-posterior (A) and lateral (B) views offer an initial assessment of the bony structures and alignment. The CT scan further enhances the visualization of the elbow in greater detail. The coronal view (C), sagittal views (D and E), and axial view (F) show a large, mildly expansile, lytic lesion noted at the distal humeral condyle abutting the articular surface.

X-ray imaging and computed tomography (CT) of the left elbow revealed an epiphyseal expansile lytic lesion in the distal humerus, causing cortical thinning with multiple areas of cortical breakthrough, with a relatively narrow zone of transition and thin intervening, enhancing separation favoring secondary ABC. There was no evidence of a periosteal reaction, pathological fracture, or soft tissue components (Figure 1).

**Figure 2:**
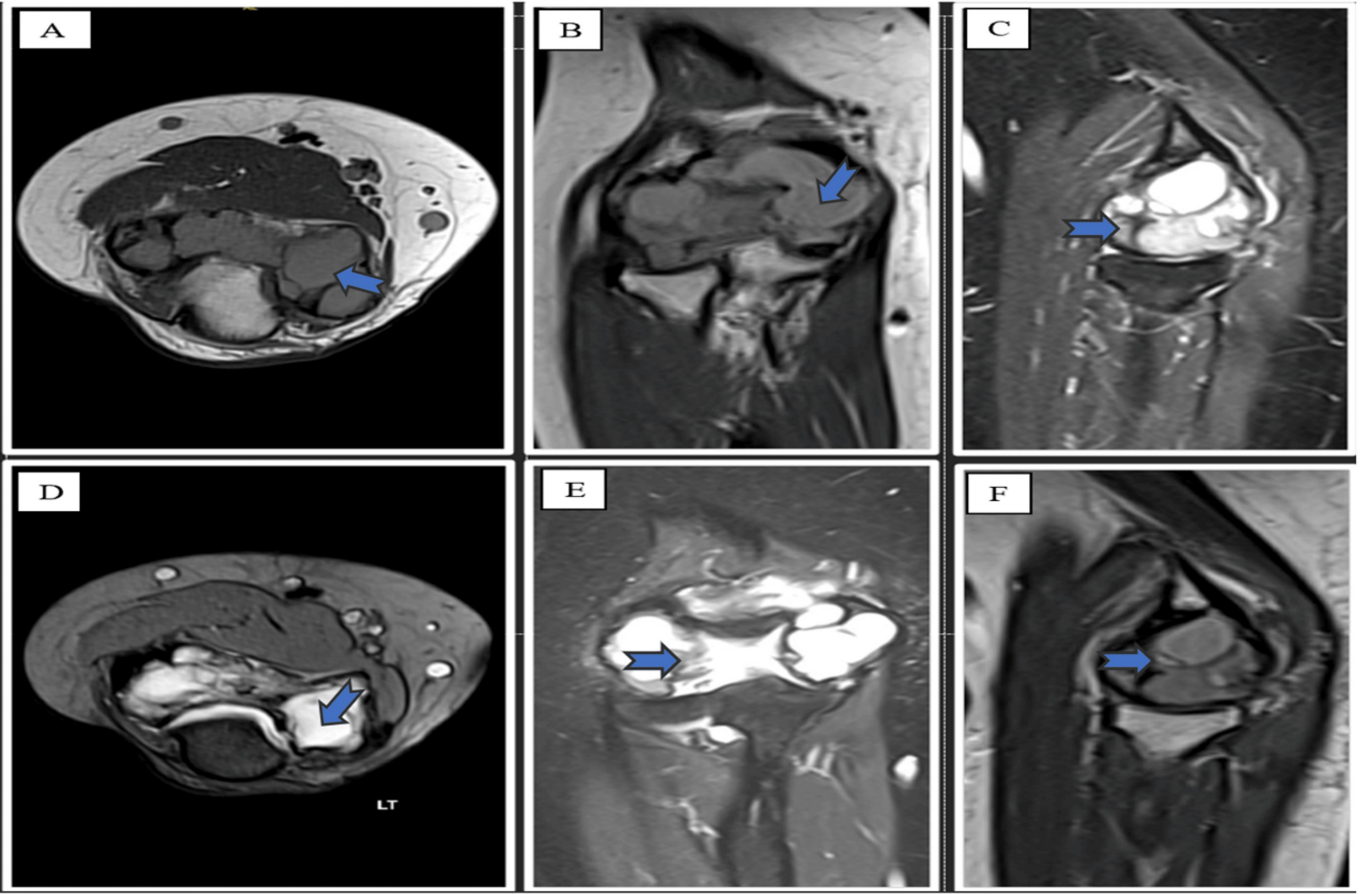
MRI demonstrated a large, eccentric, solid, and cystic lesion at the epiphysis of the left distal humerus, causing cortical breakthrough. (A) An MRI T1 axial view of the elbow. (B) An MRI T1 coronal view of the elbow. (C) The elbow's MRI T1 sagittal view. (D) An MRI T2 axial view of the elbow. (E) An MRI T2 coronal view of the elbow. (F) An MRI T2 sagittal view of the elbow. MRI: magnetic resonance imaging

Magnetic resonance imaging (MRI) revealed a large, eccentric, solid, and cystic lesion at the epiphysis of the left distal humerus, causing a cortical breakthrough. The lesion measured approximately 5.5×1.3×3.7 cm in transverse dimensions, consistent with GCTs with an associated ABC (Figure 2).

Biopsy and diagnosis

**Figure 3:**
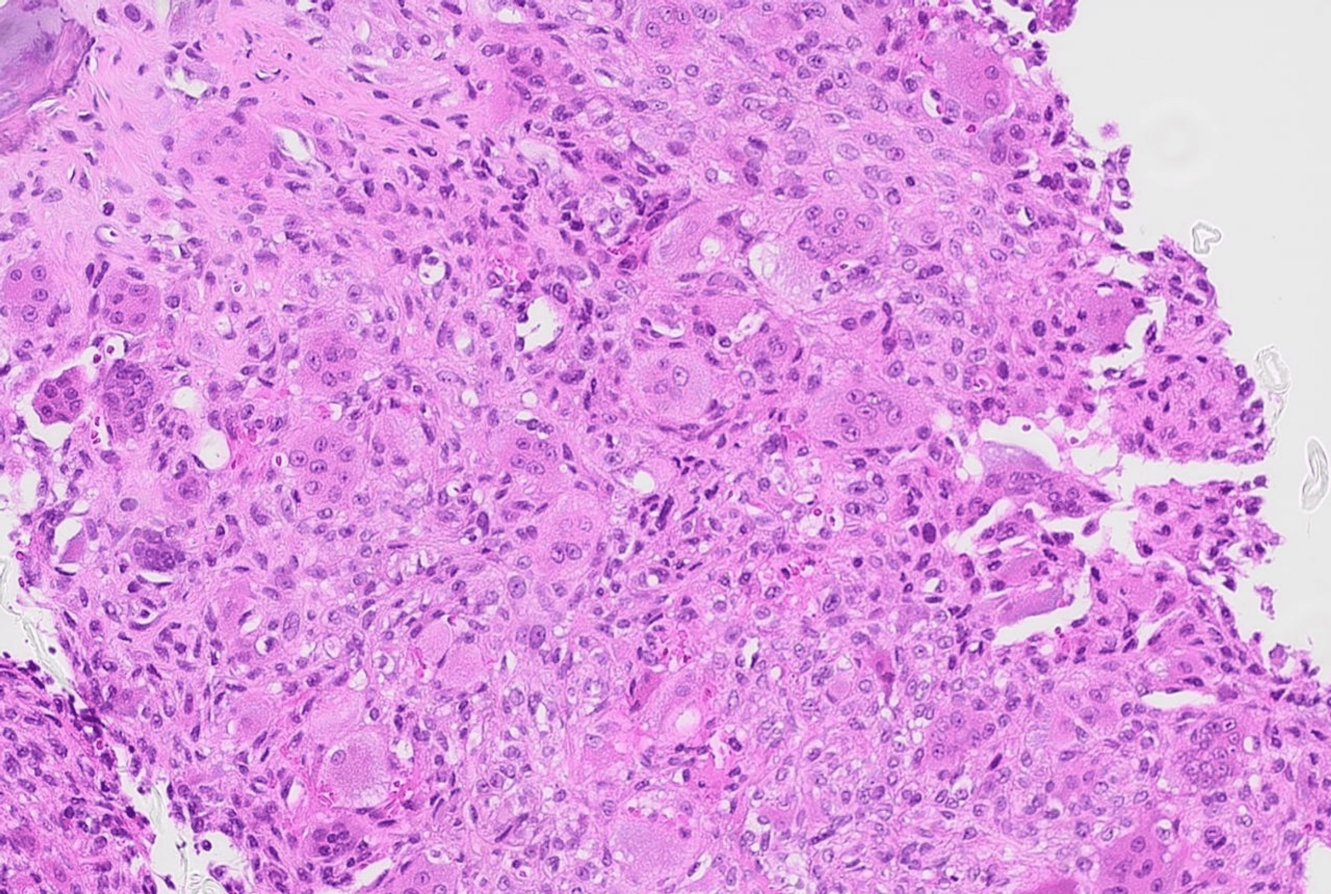
H&E stains (20×): giant cell-rich lesion composed of mononuclear cells and multinucleated giant cells.

The patient was referred to an interventional radiologist for a lesion biopsy. Biopsy results confirmed the presence of GCTs with secondary ABC. Histopathological data in Figure 3 show a giant cell-rich lesion composed of mononuclear cells and multinucleated giant cells, which are osteoclast-like cells that actively resorb the host bone through cathepsin K activity.

Surgical intervention

**Figure 4:**
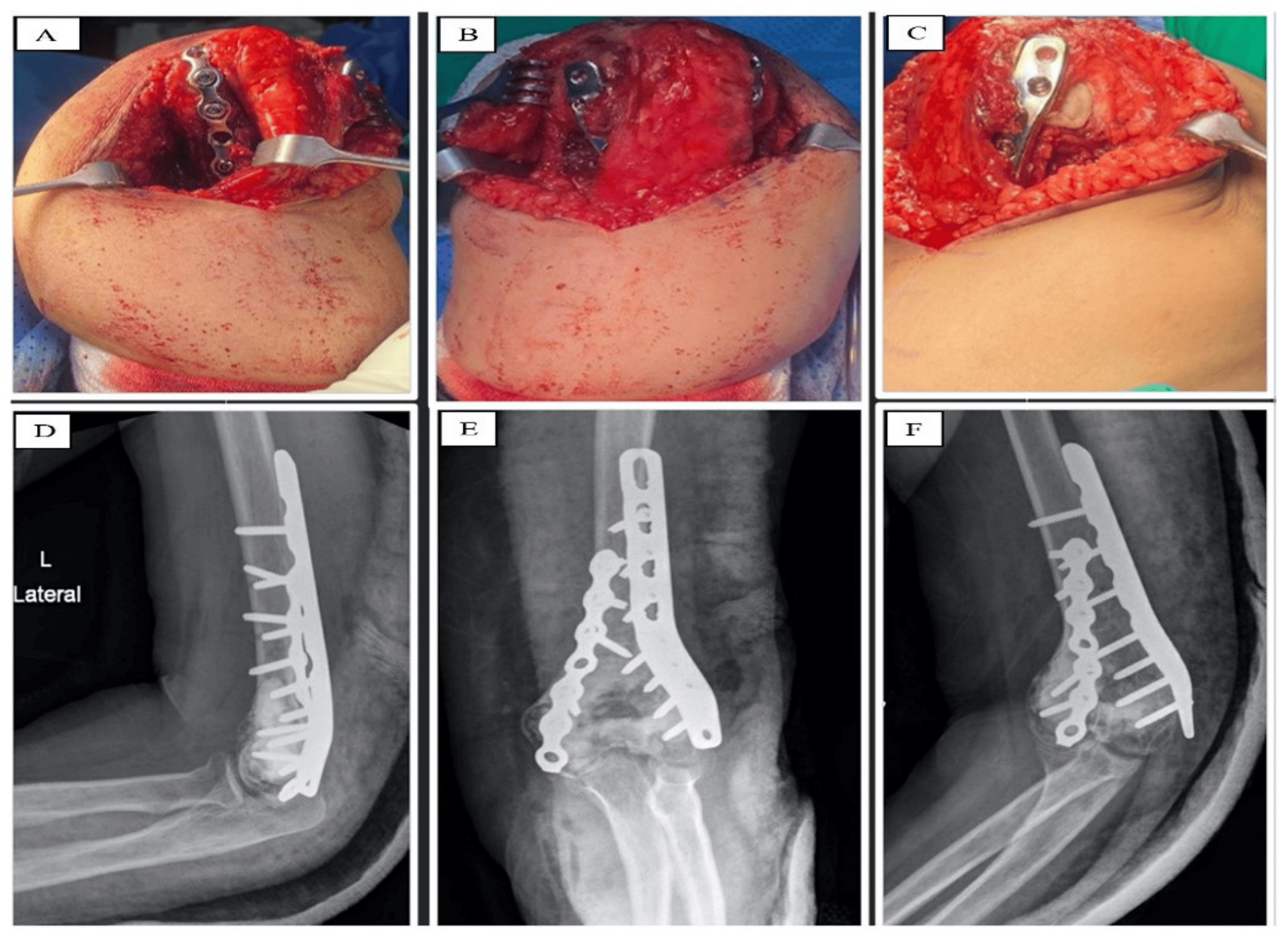
Surgical site with plates, cement application, and postoperative X-ray. (A) The surgical approach with the implant in place from the medial side. (B) The surgical approach with the implant in place from the posterior side. (C) The surgical approach with the implant and cement in place from the lateral side. (D) An X-ray lateral view of the elbow postoperatively. (E) An X-ray AP view of the elbow postoperatively. (F) An X-ray oblique view of the elbow postoperatively. AP: anterior-posterior

On July 31, 2023, the patient underwent surgical intervention, a posterior distal humerus participial Alonso-Llames, which is a posterior paratricipital approach to the distal humerus, starting with an incision centered on the junction of the middle and distal thirds of the humeral shaft until the proximal ulnar diaphysis, followed by the elevation of full-thickness flab and then making radial and ulnar window by the elevation of the triceps off the posterior humerus while preserving triceps insertion intact. A lateral dynamic compression plate and a posteromedial nine-hole reconstruction plate were applied laterally. Intralesional curettage and tissue biopsy were performed, followed by cement placement in the defect area. Ulnar nerve transposition and backslapping, which is applying a backslap cast on the skin (a slab of plaster that does not completely encircle the limb), and closure were performed (Figure 4).

Follow-up and outcome

Postoperatively, the patient received appropriate analgesia, and three doses of antibiotics as prophylaxis with a postoperative course were smooth with no complications and complete recovery. The patient received regular follow-up care to monitor her progress and to assess any signs of recurrence or complications. When writing this report, the patient was within the early postoperative period (three months), and long-term outcomes and recurrence are yet to be determined. On October 22, 2023, physical therapy/occupation therapy was started, in which the course focused on elbow muscle power with delicate motor tasks and extensive elbow range of motion strengthening exercises.

## Discussion

This case report describes the diagnosis and management of GCT with secondary ABC in the left distal humerus. The unusual location and the presence of the secondary ABC made this case particularly challenging, especially given that the primary GCT of the distal humerus is already a rare entity. The concurrence of ABC adds further complexity to both the clinical presentation and radiological picture.

The combination of GCT with secondary ABC is an uncommon pathological entity, occurring in approximately 14%-39% of GCT cases [5,6]. This association can significantly alter the radiological appearance and clinical behavior of the primary GCT, potentially complicating diagnosis and treatment planning. In our case, the presence of the secondary ABC contributed to the expansile nature of the lesion and the cortical breakthrough observed in imaging studies.

The distal humerus is an atypical location for GCT, representing only about 6% of all GCT cases [3-11]. When combined with a secondary ABC, as in our patient, the rarity of the presentation increases substantially. This uncommon location poses unique challenges for surgical management due to the complex anatomy of the elbow joint and the proximity of important neurovascular structures [7].

The etiology of GCTs has been linked to specific mutations in the *H3F3A* gene, which encodes histone H3.3 [11-14]. While this genetic aspect is crucial for understanding the pathogenesis of GCTs, it is important to note that our case presented as a primary benign form, distinct from the malignant variants of GCT that can occur [13,15]. The etiology of secondary ABC formation in GCT is not fully understood, but it is thought to be related to hemodynamic changes within the tumor [5,13].

The presence of ABC can lead to a more aggressive behavior of the lesion, with an increased risk of pathological fracture and local recurrence. In our case, the patient presented with progressive pain and functional limitations, which may have been exacerbated by the presence of the secondary ABC. It is worth noting that while the spine and skull are rarely affected by GCTs, our case in the distal humerus represents one of the infrequent occurrences in long bones outside the most common sites [13].

Diagnostic imaging plays a crucial role in identifying the combination of GCT with secondary ABC. In our patient, the CT and MRI findings were instrumental in revealing the characteristic features of both entities. The expansile nature of the lesion, the presence of fluid-fluid levels, and the enhancement pattern on MRI were suggestive of the secondary ABC component [5,6].

The treatment approach for GCT with secondary ABC requires careful consideration. While curettage and bone grafting or cementation remain the standard treatment for most GCTs, the presence of a secondary ABC may necessitate more aggressive management to reduce the risk of recurrence [11,12]. In our case, we opted for intralesional curettage, cement placement, and prophylactic fixation with plates and screws, which provided good short-term outcomes. This approach aligns with current treatment modalities that range from medical management to more invasive surgical interventions, depending on the specific characteristics of the lesion [15].

The learning points from this case include the need to consider the diagnosis of GCT with secondary ABC, even in atypical locations such as the distal humerus. The presence of secondary ABC should raise the index of suspicion for more aggressive behavior and the potential need for more extensive surgical intervention. Additionally, long-term follow-up is crucial in these cases due to the higher risk of local recurrence associated with both GCT and ABC [9,10].

This case demonstrates the challenges of successful bone tumor surgery in anatomically complex areas and highlights the importance of a thoughtful approach to planning, meticulous surgical techniques, and vigilant follow-up. The multidisciplinary approach, involving orthopedic oncologists, radiologists, and pathologists, is essential for the optimal management of these rare and complex cases.

## Conclusions

The learning points from this case are the need to consider the diagnosis of GCT with a high index of suspicion, even in atypical locations, and the necessity of a third-generation multimodal integrated approach to definitive treatment. The surgical procedure included a posterior distal approach to the humerus, the protection and mobilization of the ulnar nerve, plating for the stabilization of the defect after partial distal humeral excision, intralesional resection, and adequate cement placement in terms of short-term and immediate outcomes. However, long-term follow-up is necessary and is also a limitation in our case because GCT is known for its local invasiveness and high recurrence rates, especially in anatomically complex areas, such as the distal humerus. This case demonstrates the challenge of successful bone tumor surgery and the importance of a thoughtful approach to planning, careful surgical techniques, and vigilant follow-up.
